# Positive response to trastuzumab deruxtecan in a patient with HER2-mutant NSCLC after multiple lines therapy, including T-DM1: a case report

**DOI:** 10.3389/fonc.2023.1268260

**Published:** 2024-01-18

**Authors:** Junzhu Xu, Bo He, Yunan Wang, Mengjia Wu, Yanyi Lu, Zixuan Su, Shujun Liu, Fengmin Yin, Jian-Guo Zhou, Wei Hu

**Affiliations:** Department of Oncology, The Second Affiliated Hospital of Zunyi Medical University, Zunyi, Guizhou, China

**Keywords:** HER2 mutation, non-small cell lung cancer, chemotherapy, target-therapy, T-DXd, ADCS

## Abstract

Human epidermal growth factor 2 (*HER2*) mutations are uncommon in non-small cell lung cancer (NSCLC), and the lack of established, effective, targeted drugs has resulted in a persistently poor prognosis. Herein, we report the case of a non-smoking, 58-year-old man diagnosed with lung adenocarcinoma (cT3N0M1c, stage IVB) harboring a *HER2* mutation (Y772_A775dupYVMA) and PD-L1 (-). The patient’s Eastern Cooperative Oncology Group performance status (PS) score was assessed as 1. He commenced first-line treatment with chemotherapy, followed by immuno-chemotherapy, and with disease progression, he received HER2-targeted therapy and chemotherapy with an anti-angiogenic agent. However, HER2-targeted therapy, including pan-HER tyrosine kinase inhibitors (afatinib, pyrotinib, and pozitinib) and antibody–drug conjugate (T-DM1), produced only stable disease (SD) as the best response. After the previously described treatment, primary tumor recurrence and multiple brain metastases were observed. Despite the patient’s compromised overall physical condition with a PS score of 3-4, he was administered T-DXd in addition to whole-brain radiotherapy (WBRT). Remarkably, both intracranial metastases and primary lesions were significantly reduced, he achieved a partial response (PR), and his PS score increased from 3-4 to 1. He was then treated with T-DXd for almost 9 months until the disease again progressed, and he did not discontinue the drug despite the occurrence of myelosuppression during this period. This is a critical case as it exerted an effective response to T-DXd despite multiple lines therapy, including T-DM1. Simultaneously, despite the occurrence of myelosuppression in the patient during T-DXd, it was controlled after aggressive treatment.

## Background

Lung cancer is the most common cause of cancer-related death worldwide (1). Non-small cell lung cancer (NSCLC) is the predominant type of lung cancer, accounting for 85% of all cases. However, human epidermal growth factor 2 (HER2, erbB-2/neu) mutations occur in only 2–4% of NSCLC patients, more commonly in women, never-smokers, and adenocarcinoma (2). HER2 is a member of the ErbB receptor tyrosine kinase family, and its mutations in NSCLC are predominantly an in-frame insertion of exon 20 into the tyrosine kinase structural domain, including Y772_A775dupYVMA, G778_P780dup, and G776delinsVC (G776delinsLC) (3, 4), which is associated with poor overall survival (OS) of only 18–21 months (5). Although HER2 can be regarded as a therapeutic target, the efficacy of targeted therapy in HER2-mutant NSCLC has been disputed. Therefore, the standard first-line treatment remains a reference for advanced “driverless” NSCLC. Antibody-drug conjugates (ADCs) are novel antitumor agents that combine the high specificity of antibody targeting with potent cytotoxic drugs (6). For cancer patients with HER2 mutation or amplification, particularly breast and gastric cancer, numerous clinical trials have demonstrated the promise of ADCs as an effective treatment strategy. Nevertheless, research into potentially effective treatments for advanced NSCLC with HER2 mutations is still ongoing.

**Figure 1:**
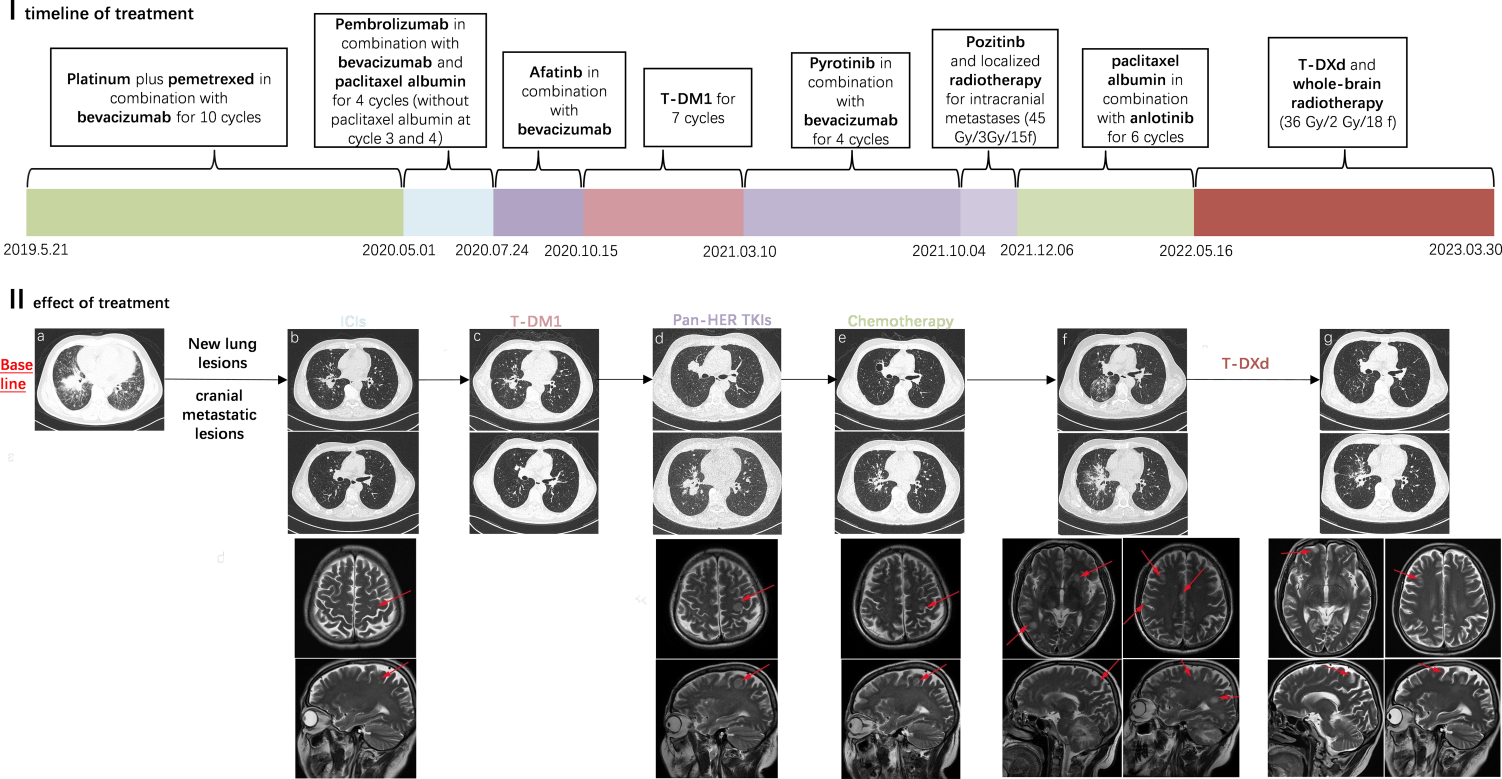
I (x-coordinate is the time, the unit is months): timeline diagram of the case receiving multiple lines of treatment; the PFS during this treatment period is expressed in length. II: Efficacy of treatment as demonstrated by chest CT and cranial MRI; **(a)** Primary lesions in the lungs **(b)** After treatment with ICIs, new lesions in the lungs and cranial metastatic lesions were observed **(c)** During treatment with T-DM1 **(d)** Disease progression after multiple lines pan-HER TKI therapy **(e)** Significant reduction in lung and brain lesions during treatment with re-challenge chemotherapy combination **(f)** Disease progression and multiple metastases in the brain **(g)** Radiotherapy was given for the cranial metastases, and subsequently in treatment with T-Dxd.

Herein, we present a patient with an overall survival (OS) of 46.5 months who underwent chemotherapy, immune checkpoint inhibitors (ICIs), anti-angiogenesis agents (bevacizumab and anlotinib), pan-HER tyrosine kinase inhibitors (afatinib, pyrotinib, and poziotinib), and ADCs (ado-trastuzumab emtansine [T-DM1], trastuzumab deruxtecan [T-DXd]), and attempt to provide new evidence for effective treatment of patients with HER2-mutant cancer (**Figure 1-I**).

## Case presentation

A 58-year-old male never-smoker was found to have an occupying lesion in the right hilar lung on chest computed tomography (CT) during a medical examination on 12.05.2019 (**Figure 1-IIa**). A biopsy was performed, and pathology revealed adenocarcinoma. The patient had diabetes, but it was under control. He then completed a systemic evaluation, which revealed pericardial and sacrococcygeal metastases. Next-generation sequencing identified a HER2 mutation (p.Y772_A775dupYVMA) and a *BRCA2* germline mutation with PD-L1 (-) and microsatellite stability. He was ultimately diagnosed with advanced-stage lung adenocarcinoma (cT3N0M1c [pericardial and sacral metastases], stage IVB) with an Eastern Cooperative Oncology Group performance status (ECOG PS) of 1. The pericardial effusion was drained, and the patient was administered one cycle of cisplatin. Subsequently, he underwent six cycles of first-line chemotherapy (pemetrexed plus cisplatin plus bevacizumab) and four cycles of maintenance therapy. A partial response (PR) was achieved at each efficacy evaluation, and progression-free survival (PFS) from first-line treatment was 11.3 months. In May 2020, with progressive disease (PD), the subject received a second-line immuno-chemotherapy regimen (paclitaxel albumin plus pembrolizumab plus bevacizumab) and was deemed to have achieved PR after two cycles. However, he discontinued paclitaxel albumin owing to unbearable bone pain. Strikingly, after two cycles, new frontal lobe and lung metastases were observed (**Figure 1-IIb**). Based on the HER2 mutation, he then received four anti-HER2 therapies, including ADCs (afatinib, T-DM1, pyrotinib, and poziotinib) for each relapse at recurrence (**Figures 1-IIc, d**). During this period, the patient underwent stereotactic radiotherapy (45 Gy/3 Gy/15 fractions) for significant enlargement of the intracranial metastasis (**Figure 1-IId**). Subsequently, a chest CT revealed an increase in bilateral pulmonary nodules. He was treated with anlotinib plus paclitaxel albumin for six cycles. After the second cycle, CT revealed a reduction in the size of the nodules in both lungs, with some lesions exhibiting cavity formation (**Figure 1-IIe**).

On 16/05/2022, significantly enlarged primary foci and multiple brain metastases were observed (**Figure 1-IIf**). The patient subsequently underwent whole-brain radiotherapy (36 Gy/2 Gy/18 f). Despite the previous treatment with T-DM1 and its poor efficacy, he chose to try T-DXd (400mg q3w) with a poor PS of 3-4. Interestingly, the patient responded to T-DXd even after heavy treatment and achieved PR (**Figure 1-IIg**), showing a significant improvement in PS (1–2). He was treated with T-DXd for almost 9 months before his disease progressed again, and he experienced neutropenia of grade 4 caused by T-DXd, which improved after supportive care. The patient died on 30.03.2023, having achieved an OS of 46.5 months.

## Discussion and conclusion

HER2 mutations are uncommon in advanced-stage NSCLC with poor prognosis, and the current standard treatment still refers to the National Comprehensive Cancer Network (NCCN) (7) recommended driverless NSCLC using platinum-pemetrexed plus bevacizumab or pembrolizumab. Also, a European EUHER2 cohort reported that in first-line treatment, PFS was 6 months and 4.8 months for chemotherapy and HER2-targeted therapy, respectively (8). In our case, we chose pemetrexed plus cisplatin plus bevacizumab as first-line treatment, and the patient was evaluated as PR during treatment with a PFS of 11.3 months. Additionally, the KEYNOTE-189 trial reported significant improvements in PFS and OS in chemotherapy-naive patients with non-squamous NSCLC, regardless of their PD-L1 expression status, using immuno-combination therapy (9). In this study, our patient received second-line albumin paclitaxel, bevacizumab, and pembrolizumab, and unfortunately, his PFS was only 2.8 months, similar to that reported in the IMMUNOTARGET retrospective study of HER2-mutant NSCLC receiving immunotherapy (10). Notably, although PR was achieved at the first evaluation, disease progression occurred after the last two cycles without albumin-paclitaxel due to bone pain. Whether disease progression is related to the discontinuation of chemotherapy or the ineffectiveness of immunotherapy remains inconclusive. However, in the first two lines of treatment, we noticed that this patient appeared to have a better response to chemotherapy. In addition, it was confirmed that afatinib and pirotinib had similar mPFS and produced responses in patients with HER2-mutant NSCLC with or without prior treatment (11–13). Also, the ZENITH20-2 trial demonstrated that poziotinib had antitumor activity in previously treated patients with HER2 exon 20 insertion NSCLC. This patient was treated with multiple pan-HER TKIs (afatinib, pyrotinib, and pozitinib) and ADC (T-DM1) based on the *HER2* mutation, but neither reached an objective response. For T-DM1, the duration of response (DoR) in our case was similar to the lung cancer cohort in the phase 2 basket study (14). Our multiple treatment attempts have failed at this point. According to the ALTER0303 trial, the mPFS of the anlotinib group in advanced NSCLC beyond third-line treatment was 5.4 months; thus, we chose conventional treatment plus anlotinib (15). Every efficacy evaluation showed PR, with a PFS of 5.7 months. In this case, we chose multiple lines therapy including chemotherapy, immunotherapy, pan-HER TKIs, and ADC, and despite the chemotherapy used in front-line and back-line, which both achieved PR and contributed to delayed progression, the disease still inevitably progressed.

With the previous multiple lines treatment having failed, the DESTINY series of studies (**Table 1**), which reported the positive efficacy of third-generation ADC (T-DXd) in HER2-altered breast and gastric cancer, has given us a new direction for treatment options (16, 17). T-DXd is a novel *HER2*-targeted ADC that consists of trastuzumab and a topoisomerase I inhibitor. Subsequently, in Destiny-Lung01 by Li et al., previously treated HER2-mutant NSCLC patients receiving T-DXd (6.4 mg/kg) had an mPFS of 8.2 months and a median overall survival of 17.8 months, while the most common drug-related adverse event of grade 3 or higher was neutropenia, and drug-related interstitial lung disease occurred in 26% of patients (18). The pivotal findings from the Destiny-Lung02 study for T-DXd dose exploration significantly contributed to the subsequent FDA approval of T-DXd on 11/08/2022 for adults with HER2-mutant NSCLC who have experienced disease progression following systemic platinum-based therapy (19). Our patient received T-DXd in failure of T-DM1 treatment and a poor PS score (3-4) and maintained PFS for almost 9 months. The best response was assessed as PR, and the ECOG PS score improved to 1-2, which is similar to a case that reported an objective response and ECOG PS score improvement following treatment with T-DXd in poor PS (20). However, both Destiny-Lung01 and Destiny-Lung02 excluded patients with a PS score >2 or who had previously received T-DM1. Prior to this, only the ‘DESTINY-Breast’ clinical trial series reported a significant delay in disease progression with T-DXd in HER2-positive breast cancer patients who had previously received T-DM1 (17, 21). Our case suggests that the efficacy and safety of T-DXd were maintained in patients with advanced NSCLC who had previously received multiple lines therapy, despite the failure of T-DM1, and with poor PS scores. Although at that time we chose to administer T-DXd at a dose of 6.4 mg/kg according to Destiny-lung01, the recent Destiny-lung02 clinical trial demonstrated the efficacy of low-dose T-DXd in treating HER2-mutant NSCLC with a lower incidence of grade 3 or higher adverse events than the high-dose group (22). In addition, we caution clinicians about the four-degree myelosuppression caused by T-DXd. Based on the limitations of individual cases, the efficacy and safety of T-DXd in this group of patients still require further clinical case accumulation for validation.

**Table 1:** Several trials and cases of ADC.

Study/case report	Sample size	Population	Best response	PFS (m)	ORR n (%)	Duration of response(m)
Ph II, DESTINY- Gastric01study (NCT03329690)	119	Progression on and after ≥ 2 prior regimens	CR (1)	5.6	61/119 (51.3)	12.5
Ph Il, DESTINY- Breast01study (NCTO3248492)	184	Previously treated with T-DM1	CR (11)	16.4	112/184 (60.9)	14.8
Ph II, DESTINY- Lung01study (NCTO3505710)	91(cohort 2)	Previously not treated with HER2-targeted therapies (except for pan-HER TKls); ECOG PS of 0 to 1	CR (11)	8.2	50/91(50)	10.6
Ph II, DESTINY- Lung02study (NCT04644237)	52(cohort of 5.4mg/kg)	Previously not treated with T-DM1, ECOG PS of 0 to 1	CR (1)	/	27/52(53.8)	/
Case report	/	Progression after 5 lines of chemotherapy, ECOG PS of 3	PR	/	/	6(Ongoing, ECOG PS of 1)

This patient had both HER2 and *BRCA2* germline mutations. Our therapeutic work has focused on standard treatment for driverless advanced NSCLC in addition to treatment for the HER2 gene mutation. Notably, the patient achieved a positive response with T-DXd after T-DM1 resistance demonstrated promising survival benefits. The efficacy of T-DXd on T-DM1-resistant HER2-positive cancer cells has been suggested to be related to the elevated expression of ABCC2 (MPR2) and ABCG2 (BCRP) (23). Furthermore, for lung cancer patients with BRCA germline mutations, BRCA inhibitors in combination with chemotherapeutic agents are emerging as a viable option. Taofeek et al. reported the efficacy and safety of veliparib compared to conventional platinum-based chemotherapeutic agents for the treatment of SCLC (24, 25). Lynnette et al. revealed a significant anti-tumor effect when combining olaparib and icotinib (26). Based on the above positive results of combination therapy with BRCA inhibitors in the treatment of lung cancer, more extensive research is required to ascertain whether combining ADCs with BRCA inhibitors could offer a novel and potential treatment approach for patients with concurrent HER2 and BRCA germline mutations.

In conclusion, we have reported a case with the HER2 mutation in an NSCLC patient who tried various treatments after progressing on standard therapy and achieved an OS of 46.5 months. This case is a reminder to clinicians that such patients could still benefit from T-DXd despite having poor PS scores after multiple lines therapy, including T-DM1, which still needs to be confirmed by more data and prospective clinical trials.

## Data availability statement

The data are not available for public access due to patient privacy concerns but are available from the corresponding author upon reasonable request.

## Ethics statement

This study complied with the tenets of the Declaration of Helsinki. It was conducted following formal approval by the Ethics Committee of the Second Affiliated Hospital of Zunyi Medical University (YXLL(KY-R)-2021-012). Written informed consent was obtained from the patient for the publication of any identifiable images or data contained herein.

## Author contributions

JX: Writing – original draft. BH: Writing – review & editing. YW: Writing – review & editing. MW: Writing – review & editing. YL: Writing – review & editing. ZS: Writing – review & editing. SL: Writing – review & editing. FY: Writing – review & editing. JZ: Funding acquisition, Writing – review & editing. WH: Funding acquisition, Writing – review & editing.
